# Supplementary material for: Multidimensional analysis of register variation in English translations of Shijing
Source: PLoS One. 2025 Sep 18;20(9):e0332503. doi: 10.1371/journal.pone.0332503 (PMC12445467; doi:10.1371/journal.pone.0332503)
Supplement: Attachment — Submitted filename: PONE-D-25-26290_reviewer.pdf [file pone.0332503.s001.pdf]

PLOS ONE

A Multi-dimensional Analysis of Register Variations in the English Translations of Shijing

--Manuscript Draft--

**Manuscript Number:**	PONE-D-25-26290
**Article Type:**	Research Article
**Full Title:**	A Multi-dimensional Analysis of Register Variations in the English Translations of Shijing
**Short Title:**	Register Variations in the English Translations of Shijing
**Corresponding Author:**	Guangwei Li<br>University of South China School of Languages and Literature<br>Qiqihar, --- Select One --- CHINA
**Keywords:**	Multidimensional analysis; Register Variation; Arthur Waley; Ezra Pound; Shijing; Chinese classical poetry; stylistic analysis
**Abstract:**	This research, employing Douglas Biber's multidimensional analysis, compares the registers of Arthur Waley's and Ezra Pound's translations of 诗经 (Shijing), examining the factors that contribute to their differences. The key findings are as follows: (1) Waley's translation corresponds to the "involved persuasion" register, characterized by high interactivity and extensive informational elaboration, while Pound's translation corresponds to the "general narrative exposition" register, emphasizing informativeness and narrativity; (2) The interactivity in Waley's translation is primarily driven by the use of analytic negation, first-person pronouns, and modal verbs, whereas the elaboration is attributed to the frequent use of demonstrative pronouns. In contrast, Pound's translation exhibits strong informativeness due to the frequent use of nouns and prepositional phrases, while its narrativity is shaped by synthetic negation and public verbs; (3) Waley's approach prioritizes an accurate reflection of ancient Chinese society and the preservation of cultural heterogeneity, whereas Pound's translation focuses on didacticism, emotional energy, and precision. The differences in the translators' ideologies and poetic philosophies are the primary factors accounting for the register variations in their translations.
**Order of Authors:**	Baohu Li
	Guangwei Li
**Additional Information:**	
**Question**	**Response**
**Financial Disclosure**<br><br>Enter a financial disclosure statement that describes the sources of funding for the work included in this submission. Review the submission guidelines for detailed requirements. View published research articles from *PLOS ONE* for specific examples.<br><br>This statement is required for submission and **will appear in the published article** if the submission is accepted. Please make sure it is accurate.	Yes

Powered by Editorial Manager® and ProduXion Manager® from Aries Systems Corporation

**Funded studies**<br>Enter a statement with the following details:<br>• Initials of the authors who received each award<br>• Grant numbers awarded to each author<br>• The full name of each funder<br>• URL of each funder website<br>• Did the sponsors or funders play any role in the study design, data collection and analysis, decision to publish, or preparation of the manuscript?<br><br>Did you receive funding for this work?	
Please add funding details.<br>as follow-up to "**Financial Disclosure**<br><br>Enter a financial disclosure statement that describes the sources of funding for the work included in this submission. Review the submission guidelines for detailed requirements. View published research articles from *PLOS ONE* for specific examples.<br><br>This statement is required for submission and **will appear in the published article** if the submission is accepted. Please make sure it is accurate.<br><br>**Funded studies**<br>Enter a statement with the following details:<br>• Initials of the authors who received each award<br>• Grant numbers awarded to each author<br>• The full name of each funder<br>• URL of each funder website<br>• Did the sponsors or funders play any role in the study design, data collection and analysis, decision to publish, or preparation of the manuscript?<br><br>Did you receive funding for this work?"	On the English Translation, Dissemination and Reception of Book of Poetry (Shijing) from the Perspective of Digital Humanities (22BYY039)
Please select the country of your main research funder (please select carefully as in some cases this is used in fee calculation).	CHINA - CN

as follow-up to "**Financial Disclosure**<br><br>Enter a financial disclosure statement that describes the sources of funding for the work included in this submission. Review the submission guidelines for detailed requirements. View published research articles from *PLOS ONE* for specific examples.<br><br>This statement is required for submission and **will appear in the published article** if the submission is accepted. Please make sure it is accurate.<br><br>**Funded studies**<br>Enter a statement with the following details:<br>• Initials of the authors who received each award<br>• Grant numbers awarded to each author<br>• The full name of each funder<br>• URL of each funder website<br>• Did the sponsors or funders play any role in the study design, data collection and analysis, decision to publish, or preparation of the manuscript?<br><br>Did you receive funding for this work?"	
**Competing Interests**<br><br>Use the instructions below to enter a competing interest statement for this submission. On behalf of all authors, disclose any competing interests that could be perceived to bias this work—acknowledging all financial support and any other relevant financial or non-financial competing interests.<br><br>This statement is required for submission and **will appear in the published article** if the submission is accepted. Please make sure it is accurate and that any funding sources listed in your Funding Information later in the submission form are also declared in your Financial Disclosure statement.	The authors have declared that no competing interests exist.

View published research articles from *PLOS ONE* for specific examples.<br><br>**NO authors have competing interests**<br><br>Enter: *The authors have declared that no competing interests exist.*<br><br>**Authors with competing interests**<br><br>Enter competing interest details beginning with this statement:<br><br>*I have read the journal's policy and the authors of this manuscript have the following competing interests: [insert competing interests here]*<br><br>* typeset	
**Ethics Statement**<br><br>Enter an ethics statement for this submission. This statement is required if the study involved:<br><br>• Human participants<br>• Human specimens or tissue<br>• Vertebrate animals or cephalopods<br>• Vertebrate embryos or tissues<br>• Field research<br><br>Write "N/A" if the submission does not require an ethics statement.<br><br>General guidance is provided below. Consult the submission guidelines for detailed instructions. **Make sure that all information entered here is included in the Methods section of the manuscript.**	N/A, the submission does not require an ethics statement

**Format for specific study types**<br><br>**Human Subject Research (involving human participants and/or tissue)**<br>• Give the name of the institutional review board or ethics committee that approved the study<br>• Include the approval number and/or a statement indicating approval of this research<br>• Indicate the form of consent obtained (written/oral) or the reason that consent was not obtained (e.g. the data were analyzed anonymously)<br><br>**Animal Research (involving vertebrate animals, embryos or tissues)**<br>• Provide the name of the Institutional Animal Care and Use Committee (IACUC) or other relevant ethics board that reviewed the study protocol, and indicate whether they approved this research or granted a formal waiver of ethical approval<br>• Include an approval number if one was obtained<br>• If the study involved *non-human primates*, add *additional details* about animal welfare and steps taken to ameliorate suffering<br>• If anesthesia, euthanasia, or any kind of animal sacrifice is part of the study, include briefly which substances and/or methods were applied<br><br>**Field Research**<br><br>Include the following details if this study involves the collection of plant, animal, or other materials from a natural setting:<br>• Field permit number<br>• Name of the institution or relevant body that granted permission	
**Data Availability**<br><br>Authors are required to make all data underlying the findings described fully available, without restriction, and from the time of publication. PLOS allows rare exceptions to address legal and ethical concerns. See the PLOS Data Policy and FAQ for detailed information.	Yes - all data are fully available without restriction

A Data Availability Statement describing where the data can be found is required at submission. Your answers to this question constitute the Data Availability Statement and **will be published in the article**, if accepted.<br><br>**Important:** Stating 'data available on request from the author' is not sufficient. If your data are only available upon request, select 'No' for the first question and explain your exceptional situation in the text box.<br><br>Do the authors confirm that all data underlying the findings described in their manuscript are fully available without restriction?	
**Describe where the data may be found in full sentences. If you are copying our sample text, replace any instances of XXX with the appropriate details.**<br><br>• If the data are **held or will be held in a public repository**, include URLs, accession numbers or DOIs. If this information will only be available after acceptance, indicate this by ticking the box below. For example: *All XXX files are available from the XXX database (accession number(s) XXX, XXX.*).<br>• If the data are all contained **within the manuscript and/or Supporting Information files**, enter the following: *All relevant data are within the manuscript and its Supporting Information files.*<br>• If neither of these applies but you are able to provide **details of access elsewhere**, with or without limitations, please do so. For example:<br><br>*Data cannot be shared publicly because of [XXX]. Data are available from the XXX Institutional Data Access / Ethics Committee (contact via XXX) for researchers who meet the criteria for access to confidential data.*<br><br>*The data underlying the results presented in the study are available from (include the name of the third party*	The data underlying the results presented in the study are available from https://github.com/zijinmingyue123/A-Multi-dimensional-Analysis-of-Register-Variations-in-the-English-Translations-of-Shijing.git

and contact information or URL).<br>• This text is appropriate if the data are owned by a third party and authors do not have permission to share the data.<br><br>* typeset	
Additional data availability information:	

Manuscript Click here to access/download;Manuscript;Manuscript.docx

Title Page

Title:

A Multi-dimensional Analysis of Register Variations in the English Translations of *Shijing*

Names of authors:

Baohu Li[1], Guangwei LI[1*]

Affiliations and addresses:

[1] University of South China, School of Languages and Literature, 421000, China.

***Corresponding author**:

Guangwei Li

University of South China, School of Languages and Literature

E-mail: hu0701002@126.com

A Multi-dimensional Analysis of Register Variations in the English Translations of *Shijing*

Abstract: This research, employing Douglas Biber's multidimensional analysis, compares the registers of Arthur Waley's and Ezra Pound's translations of *诗经* (*Shijing*), examining the factors that contribute to their differences. The key findings are as follows: (1) Waley's translation corresponds to the "involved persuasion" register, characterized by high interactivity and extensive informational elaboration, while Pound's translation corresponds to the "general narrative exposition" register, emphasizing informativeness and narrativity; (2) The interactivity in Waley's translation is primarily driven by the use of analytic negation, first-person pronouns, and modal verbs, whereas the elaboration is attributed to the frequent use of demonstrative pronouns. In contrast, Pound's translation exhibits strong informativeness due to the frequent use of nouns and prepositional phrases, while its narrativity is shaped by synthetic negation and public verbs; (3) Waley's approach prioritizes an accurate reflection of ancient Chinese society and the preservation of cultural heterogeneity, whereas Pound's translation focuses on didacticism, emotional energy, and precision. The differences in the translators' ideologies and poetic philosophies are the primary factors accounting for the register variations in their translations.
Keywords: Multidimensional analysis; Register Variation; Arthur Waley; Ezra Pound; *Shijing*; Chinese classical poetry; stylistic analysis

1. Introduction

Chinese classical literature boasts a rich heritage, with classical poetry being one of the earliest forms translated and studied abroad, significantly influencing Western societies (Wang et al., 2021: 67). *诗经* (*Shijing*) is the earliest anthology of ancient Chinese poetry. In 1736, Richard Brookes translated the French Description de la Chine into English, including eight *Shijing* poems, such as *Tian Zuo* (*天作*) and *Huang Yi* (*皇矣*) (Ma and Ren, 2003: 47). Since then, the English translation of the *Shijing* has a history of nearly three centuries (Li, 2021: 3). Among these, Arthur Waley's 1937 translation deviated from traditional arrangements, reorganizing the poems through a cultural anthropology lens. Similarly, Ezra Pound's 1954 translation is distinguished by its imagist style, often included in modern poetry anthologies as a creative work in its own right (Li, 2021: 50).

The term "register" refers to the suitability of vocabulary and sentence structure for a particular style (Wang and Ding 1987: 414), representing a cluster of linguistic features that tend to co-occur more frequently than would be expected by chance (Halliday, 1988: 162). In translating *Shijing* into English, Arthur Waley and Ezra Pound may make distinct choices in vocabulary and sentence structure, which in turn shaped the registers of their translations. Comparing the registers of Waley's and Pound's translations not only reveals the stylistic characteristics of each but also provides insight into the factors influencing register formation in English renditions of *Shijing*.

2. Previous Literature on Register in Translation Studies

The advancements in natural language processing (NLP) have made corpus-based methods a key tool in poetry research. Concurrently, the study of register—the appropriateness of language in specific contexts—has garnered significant attention in translation studies, with a focus on specialized domains such as academic texts, legal discourse, maritime language, and political texts. Notable studies in the areas include Garrote Pilar Robles (2021), who examines structural features in academic emails from Spanish and Italian students; Chen Meishan (2021), who analyzes the lexico-grammatical aspects of courtroom discourse; and Poole Robert (2021), who investigates stance adverbs in U.S. Federal Courts of Appeals opinions; Isobelle Clarke (2022), who applies a short-text version of Multi-Dimensional Analysis (MDA) to a corpus of English tweets, uncovering common patterns of linguistic variation. Additional research by Ryoo Mi-Lim and Cao Di (2021) compares maritime English with academic texts, while Jack Grieve (2023) applies stylometric analysis to distinguish registers in political texts. Research on register has also explored business, speech, and literary texts. For instance, Jiang Jinlin and Xu Jiajin (2015) use MDA to differentiate business English from general English, while Yang Linlin and Zhong Lingli (2018) apply MDA to committee hearing English. Studies on speeches (Wang Wei, 2021), shareholder letters (Hu Chunyu and Tan Jinlin, 2020), and literary translations (Zhao Chaoyong, 2020a) further contribute to understanding register across genres.

However, limited attention has been given to the language of poetry, particularly Chinese classical poetry with numerous translations. The register differences in these translations, shaped by the translators' linguistic choices, remain underexplored. A more systematic study of these differences would shed light on the stylistic features of translations and provide objective insights into their formation.

3. Research Design

3.1 Research Questions

This research aims to address the following questions:

1. Do Arthur Waley’s and Ezra Pound’s translations of *Shijing* belong to the same register? If not, what are the differences in their registers?

2. If the two translations belong to different registers, what linguistic features account for these differences?

3. If the two translations belong to different registers, what are the main factors influencing these differences?

3.2 Research Objects

The research focuses on two translations: (1) *The Book of Songs*, translated by British sinologist Arthur Waley and published by Grove Press in 1996; and (2) *The Classic Anthology Defined by Confucius*, translated by American poet Ezra Pound and

published by Faber and Faber Ltd. in 1954. To ensure comparability between Waley's and Pound's translations, the titles in Waley's version have been excluded, as Pound's translation omits titles. The total character counts for Waley's and Pound's translations are 36,528 and 44,170, respectively.

3.3 Tools and Methods

This study employs MDA and follows the approach outlined by Zhao Chaoyong (2020b) for analyzing translator styles. MDA, developed by Douglas Biber, uses factor analysis and cluster analysis to identify and calculate the frequency and standardized frequency per thousand words of sixty-seven linguistic features. These features are grouped into seven dimensions based on their co-occurrence within specific registers, resulting in factor scores or dimension values, which may be either positive or negative. This method quantifies linguistic features to categorize registers. However, due to limited data in Dimension 7 and its lack of comparability with the other six dimensions, it is typically excluded in practical applications (Zhao, 2020b: 68-69). Additionally, SPSS statistical analysis software was used to conduct significance tests on the dimension scores and Z-values calculated by MAT 1.3.3, along with multiple linear regression to identify key linguistic features influencing variation across register dimensions. Finally, corpus retrieval from the bilingual parallel corpus of *Shijing* was conducted to provide supporting examples.

4. Contrast of the Registers of the Two Translations of *Shijing*

4.1 Overall Contrast of the Registers

To compare the six dimensions of the registers, this study compiled Waley's and Pound's translations of *Shijing* into text files (TXT) using OCR and manual correction. The translations were then organized into 305 separate TXT documents, corresponding to the 305 poems in the original *Shijing*. These 305 English texts from each translation were imported into MAT 1.3.3 for analysis, and their average dimension values are presented in Table 1.

Table 1 Contrast of the Register Dimensions of Waley's and Pound's Translations of *Shijing*

Dimension		D1	D2	D3	D4	D5	D6	Register
Waley's Translation (N=305)	M	6.19	0.75	2.67	-3.06	-1.54	0.03	Involved persuasion
	SD	12.636	7.352	7.358	7.517	4.684	4.505	
Pound's Translation (N=305)	M	-4.34	2.53	1.73	-2.18	-2.14	-1.66	General narrative exposition
	SD	11.625	11.161	7.140	6.129	3.265	2.743	

Note: M = mean; SD = standard deviation

The results show that Waley's translation aligns with the "involved persuasion" register, characterized by strong persuasive and argumentative features, typical of text types such as interviews and impromptu speeches. In contrast, Pound's translation

falls under the "general narrative exposition" register, emphasizing information delivery through narration, with typical text types including news reports, newspaper editorials, and science fiction novels.

To compare the dimensional differences between the two translations, independent sample t-tests were conducted using SPSS, with the results presented in Table 2.

Table 2 Contrast of Dimensional Differences between Waley's and Pound's Translations

Dimension	Waley's Translation (N=305)		Pound's Translation (N=305)		Mean Deviation	t	p
	M	SD	M	SD			
**D1**	**6.19**	**12.636**	**-4.34**	**11.625**	**10.528**	**10.709**	**0.000**
**D2**	**0.75**	**7.352**	**2.53**	**11.161**	**1.113**	**-2.316**	**0.021**
D3	2.67	7.358	1.73	7.140	0.938	1.598	0.111
D4	-3.06	7.517	-2.18	6.129	0.880	-1.585	0.114
D5	-1.54	4.684	-2.14	3.265	0.603	1.845	0.066
**D6**	**0.03**	**4.505**	**-1.66**	**2.743**	**1.686**	**5.583**	**0.000**

The results reveal significant differences between Waley's and Pound's translations in dimensions 1, 2, and 6 ($p < 0.05$). Waley's translation exhibits stronger interactive features and on-line informational elaboration but weaker narrativity, while Pound's translation demonstrates higher informativeness and narrativity, but weaker on-line informational elaboration. Additionally, linguistic features in both translations were analyzed using MAT 1.3.3, with frequencies and standardized frequencies per thousand words calculated for 67 linguistic features. Independent sample t-tests were then conducted on the linguistic features associated with the three dimensions showing significant differences, as detailed in Table 3.

Table 3 Contrast of the Linguistic Features Involved in Three Dimensions with Significant Differences

Number		Factor	Waley	Pound	t	p	Difference
1		**AMP (Amplifiers)**	**-0.15**	**-0.96**	**5.752**	**0.000**	**0.804**
2		**ANDC (Independent Clause Coordination)**	**0.09**	**0.40**	**-1.984**	**0.048**	**0.314**
3		AWL (Word length)	-1.06	-0.97	-1.252	0.211	0.087
4		CAUS (Causative Adverbial Subordinators)	-0.38	-0.60	1.729	0.085	0.219
5		DEMP (Demonstrative Pronouns)	-0.35	-0.47	1.166	0.244	0.120
6		**DPAR (Discourse Particles)**	**0.43**	**-0.20**	**3.254**	**0.001**	**0.630**
7		**EMPH (Emphatics)**	**-0.19**	**-0.86**	**3.842**	**0.000**	**0.674**
8		**FPP1 (First Person Pronouns)**	**0.97**	**0.17**	**7.038**	**0.000**	**0.792**
9	D1	INPR (Indefinite Pronouns)	0.09	-0.13	1.272	0.204	0.218
10		**JJ (Attributive Adjectives)**	**-0.04**	**0.72**	**-4.689**	**0.000**	**0.756**
11		**NN (Total Other Nouns)**	**1.60**	**3.66**	**-13.359**	**0.000**	**2.053**
12		**PIN (Total Prepositional Phrases)**	**-0.82**	**-0.49**	**-2.779**	**0.006**	**0.329**
13		**PIT (Pronoun It)**	**0.56**	**-0.78**	**7.833**	**0.000**	**1.341**
14		**POMD (Possibility Modals)**	**0.44**	**-0.30**	**2.942**	**0.003**	**0.753**
15		**SPP2 (Second Person Pronouns)**	**0.45**	**0.03**	**2.854**	**0.004**	**0.419**
16		TTR (Type-token Ratio)	-6.29	-6.05	-1.137	0.256	0.235
17		**VPRT (Present Tense)**	**0.04**	**-0.67**	**8.097**	**0.000**	**0.706**

18		**XX0 (Analytic negation)**	**0.72**	**0.16**	**2.672**	**0.008**	**0.563**
19		**BEMA (Be as Main Verb)**	**-0.23**	**-1.71**	**9.472**	**0.000**	**1.478**
20		**CONT (Contractions)**	**-0.66**	**-0.33**	**-6.565**	**0.000**	**0.328**
21		**PRIV (Private Verbs)**	**-0.86**	**-0.62**	**-2.131**	**0.033**	**0.234**
22		PROD (Pro-verb Do)	-0.62	-0.57	-0.518	0.605	0.045
23		**STPR (Stranded Preposition)**	**-0.27**	**0.36**	**-2.430**	**0.016**	**0.628**
24		THATD (Subordinator That Deletion)	-0.50	-0.41	-0.950	0.342	0.096
25		**VBD (Past Tense)**	**-0.45**	**-0.65**	**2.389**	**0.017**	**0.199**
26		**PEAS (Perfect Aspect)**	**-0.30**	**-1.25**	**6.332**	**0.000**	**0.952**
27	D2	PRESP (Present Participial Clauses)	0.34	0.43	-0.341	0.734	0.093
28		**PUBV (Public Verbs)**	**-0.87**	**-0.56**	**-2.018**	**0.044**	**0.311**
29		**SYNE (Synthetic Negation)**	**1.56**	**4.74**	**-4.862**	**0.000**	**3.187**
30		**TPP3 (Third Person Pronouns)**	**0.47**	**-0.19**	**5.809**	**0.000**	**0.668**
48	D6	**DEMO (Demonstratives)**	**0.64**	**-0.93**	**6.259**	**0.000**	**1.567**
49		THVC (That Verb Complemnts)	-0.61	-0.73	0.749	0.454	0.119

The results indicate that, among the twenty-four linguistic features in dimension 1, seventeen features exhibit significant differences between Waley's and Pound's translations, representing approximately 70.83% of all linguistic features in this dimension. In dimension 2, six linguistic features were analyzed, with five showing significant differences, accounting for 83.3% of the total. In dimension 6, two linguistic features were considered, with "Demonstratives" showing a significant difference.

4.2 Contrast of the Register Dimensions

To further explore the dimensions with significant differences and examine the relationship between linguistic features and these dimensions, this study employs stepwise regression analysis on the linguistic features in dimensions 1, 2, and 6.

4.2.1 Contrast of Informational and Involved Production

Dimension 1 represents the contrast between informational and involved production, reflecting the interactivity and informativeness of texts. Higher values indicate greater interactivity and lower informativeness. This dimension includes thirty-four linguistic features, such as PRIV, TTR, VPRT, and AWL. For linguistic features that appear in multiple dimensions, if their loadings on other dimensions exceed those in the current dimension, they are excluded from the calculations for that dimension (Zhao, 2020b: 68-69). Additionally, following Biber's (1988: 93) guidelines, features with loadings below 0.35 across all dimensions are excluded from the analysis. In dimension 1, five features, including COND and PLACE, have higher loadings on other dimensions and are therefore excluded. The feature "Present Participial WHIZ Deletions" is also excluded due to factor loadings below 0.35 on all dimensions. Furthermore, as per Nini (2019), features with Z-score means below 1 are excluded from the analysis. Thus, only twenty-four linguistic features are retained for

the calculation of scores in dimension 1.

To identify the linguistic features contributing to register differences, stepwise regression analysis was performed on the twenty-four features in dimension 1 for both Waley's and Pound's translations, with the results presented in Tables 4 and 5.

Table 4 Parameters of the Stepwise Regression Models for the Top Five Variables Predicting the Scores of Dimension 1 of Waley's and Pound's Translations

Model	R	R Square	Adjusted R Square	Std. Error of the Estimate
Waley's Translation	0.797	0.635	0.629	7.69691
Pound's Translation	0.805	0.648	0.642	6.95792

Table 5 Stepwise Regression Coefficients for Dimension 1 of Waley's and Pound's Translations

Model		Unstandardized Coefficients		Standardized Coefficients	t	p
		B	Standard Error	Beta		
Waley's Translation	(Constant)	1.742	0.969		1.797	0.073
	NN	-2.379	0.257	-0.346	-9.253	0.000
	POMD	1.013	0.125	0.289	8.088	0.000
	AWL	-5.010	0.528	-0.351	-9.497	0.000
	PIN	-2.597	0.306	-0.305	-8.488	0.000
	DPAR	0.863	0.147	0.210	5.861	0.000
Pound's Translation	(Constant)	-4.240	1.246		-3.404	0.001
	NN	-2.045	0.220	-0.344	-9.285	0.000
	XXO	1.725	0.183	0.334	9.408	0.000
	FPP1	2.778	0.367	0.282	7.570	0.000
	STPR	0.672	0.095	-0.246	7.045	0.000
	TTR	-1.054	0.155	-0.239	-6.801	0.000

The five linguistic features that most significantly contribute to the distinction between informational and involved production in Waley's translation are NN, POMD, AWL, PIN, and DPAR, collectively explaining 63.5% of the variance in this dimension (R^2 = 0.635). The standardized regression equation is as follows: Dimension 1 (Waley's translation) = 1.742 - 2.379NN + 1.013POMD - 5.010AWL - 2.597PIN + 0.863DPAR. Among these features, POMD and DPAR have positive regression coefficients, whereas NN, AWL, and PIN have negative ones. This indicates that a higher frequency of possibility modals and discourse particles enhances textual interactivity, while longer average word length and greater use of nouns (excluding nominalizations and gerunds) and prepositional phrases contribute to greater informativeness. The explanatory power of the first four linguistic features exceeds 0.05 in terms of R^2 changes (while DPAR accounts for an R^2 change of 0.042), suggesting that nouns (excluding nominalizations and gerunds), possibility modals, average word length, and prepositional phrases play a more significant role in shaping the interactivity of Waley's translation than discourse particles. Further analysis based on Table 3 reveals that the p-values for AWL in both translations

exceed 0.05, indicating that this feature does not differ significantly between the two. Therefore, the three linguistic features most responsible for the higher interactivity of Waley's translation are nouns (excluding nominalizations and gerunds), possibility modals, and prepositional phrases. Nouns serve as primary carriers of referential meaning, with higher noun frequency correlating with greater information density. Possibility modals introduce the author's viewpoint or inferential stance, actively engaging readers in interpretation and reinforcing textual interactivity. Prepositional phrases facilitate information integration within the text. Table 3 further demonstrates that compared to Pound's translation, Waley's version employs significantly fewer nouns (excluding nominalizations and gerunds) and prepositional phrases but significantly more possibility modals, resulting in a more involved register.

The five linguistic features that most significantly contribute to the score of Dimension 1 in Pound's translation are NN, XXO, FPP1, STPR, and TTR, collectively explaining 64.8% of the variance in this dimension ($R^2 = 0.648$). The standardized regression equation is: Dimension 1 (Pound's translation) = -4.240 - 2.045NN + 1.725XXO + 2.778FPP1 + 0.672STPR - 1.054TTR. Among these, XXO, FPP1, and STPR have positive regression coefficients, while NN and TTR have negative ones. This indicates that a higher frequency of analytic negation, first-person pronouns, and stranded prepositions enhances textual interactivity, whereas an increased use of nouns (excluding nominalizations and gerunds) and a higher type-token ratio contribute to greater informativeness. The first three linguistic features (NN, XXO, and FPP1) show R^2 changes above 0.05, suggesting that compared to stranded prepositions and type-token ratio, nouns (excluding nominalizations and gerunds), analytic negation, and first-person pronouns play a more significant role in shaping the informativeness of Pound's translation. Table 3 reveals significant differences ($p < 0.05$) in the usage of NN, XXO, and FPP1 between Pound's and Waley's translations. Therefore, these three linguistic features—nouns (excluding nominalizations and gerunds), analytic negation, and first-person pronouns—are the primary factors contributing to the higher informativeness of Pound's translation. Analytic negation (e.g., not) functions as an alternative to synthetic negation (e.g., no, neither), often resulting in a more fragmented presentation of information and reduced informational density. First-person pronouns directly reference the speaker and are commonly used in interactive discourse (Biber 1988: 104 - 106).

Example 1

ST (Source Text): 振鹭于飞，于彼西雝。我客戾止，亦有斯容。在彼无恶，在此无斁。庶几夙夜，以永终誉。（周颂·臣工之什·振鹭）

WT (Waley's Translation): Flock the **egrets** in their **flight**, To that western **moat**. My **guest** has come, He too with like **movements**. They there find no **harm**; Of him here we shall never weary. Through the **day**, into the **night**, May he long keep **holiday**!

PT (Pound's Translation): **Egrets** to fly to this West **Moat**, **guests** at my **portal** be such **cause** for **joy**. No **hate** roots there, and here in **court** no **irk**, but as the **seeds** of **motion** are to all their **folk**, early and late cause **praise**.

This example illustrates that Pound's translation employs a higher frequency of nouns (excluding nominalizations and gerunds) than Waley's translation. The original poem is a hymn of praise for the kings of Qi and Song, descendants of the Xia and Shang dynasties, who traveled to the Zhou ancestral temple for sacrificial rites (Cheng and Jiang 2017: 721).

Waley's translation prioritizes detailed portrayal, favoring verbs and adjectives to create a dynamic reading experience, thereby enhancing textual interactivity. For instance, in *振鹭*, Waley uses verbs such as "flock" and "come" to depict the birds' movement and the guests' arrival. This choice imbues the translation with vividness and immediacy, allowing readers to visualize the scene more intuitively. Additionally, adjectives like "western" and "weary" further enrich the imagery, lending the translation a more nuanced and evocative quality.

In contrast, Pound's translation emphasizes imagery, condensing meaning through the frequent use of nouns, which contributes to its greater informativeness. As a leading figure of the Imagist movement, Pound often preserves or reinterprets the original poem's imagery to capture its essence. For example, he translates 西雝 (western high roof) as "west moat", maintaining the poem's atmospheric depth. In other cases, he expands on the imagery to convey deeper emotions, as seen in his rendering of "庶几夙夜" (perhaps day and night) as "the seeds of motion", symbolizing persistence and the aspiration for the king's enduring legacy. Linguistically, this focus on imagery results in a high concentration of nouns. In the poem's 42 characters, 13 are nouns, comprising 30.95% of the text. As concrete lexical elements, nouns encapsulate meaning and emotion, evoking direct sensory and emotional responses from readers. This noun-dense approach lends Pound's translation a free and flexible style, intensifying the poem's imagery and emotional depth.

4.2.2 Contrast of Narrative and Non-Narrative Concerns

Dimension 2 reflects the degree of narrativity in texts, with higher values indicating greater narrativity. This dimension comprises ten linguistic features: VBD, TPP3, PEAS, PUBV, SYNE, PRESP, VPRT, JJ, WZPAST, and AWL. However, VPRT, JJ, WZPAST, and AWL exhibit higher loadings in other dimensions, necessitating their exclusion. Specifically, the loadings of VPRT on Dimensions 1 and 2 are 0.86 and -0.47, respectively; JJ's loadings on Dimensions 1 and 2 are -0.47 and -0.41; WZPAST's loadings on Dimensions 2 and 5 are -0.34 and 0.40; and AWL's loadings on Dimensions 1 and 2 are -0.58 and -0.31. Positive values indicate a positive correlation, while negative values indicate a negative one. In cross-dimensional comparisons, absolute values of loadings are taken into account. Consequently, only six linguistic features contribute to the calculation of Dimension 2 scores. Stepwise regression analysis was performed in SPSS on these six features in Waley's and Pound's translations, with results presented in Tables 6 and 7.

Table 6 Parameters of the Stepwise Regression Models for the Top Five Variables Predicting the Scores of

Dimension 2 of Waley's and Pound's Translations

Model	R	R Square	Adjusted R Square	Std. Error of the Estimate
Waley's Translation	0.988	0.976	0.975	1.15580
Pound's Translation	0.997	0.995	0.994	0.82964

Table 7 Stepwise Regression Coefficients for Dimension 2 of Waley's and Pound's Translations

Model		Unstandardized Coefficients		Standardized Coefficients	t	p
		B	Standard Error	Beta		
Waley's Translation	(Constant)	-0.545	0.079		-6.863	0.000
	SYNE	1.003	0.012	0.754	81.760	0.000
	PRESP	1.005	0.023	0.399	43.679	0.000
	PEAS	0.992	0.028	0.322	35.608	0.000
	TPP3	1.173	0.040	0.268	29.319	0.000
	PUBV	0.999	0.038	0.240	26.401	0.000
Pound's Translation	(Constant)	-0.444	0.076		-5.851	0.000
	SYNE	0.991	0.005	0.890	206.911	0.000
	PRESP	0.999	0.013	0.340	79.320	0.000
	PUBV	1.026	0.024	0.187	43.202	0.000
	PEAS	1.120	0.044	0.110	25.691	0.000
	TPP3	1.005	0.044	0.099	22.672	0.000

The five linguistic features that most significantly contribute to the non-narrativity of Waley's translation are SYNE, PRESP, PEAS, TPP3, and PUBV, collectively accounting for 97.6% of the variance in this dimension ($R^2 = 0.976$). The standardized regression equation is: Dimension 2 (Waley's translation) = -0.545 + 1.003SYNE + 1.005PRESP + 0.992PEAS + 1.173TPP3 + 0.999PUBV. All regression coefficients are positive, indicating that an increased presence of synthetic negation, present participial clauses, perfect aspect verbs, third-person pronouns, and public verbs corresponds to higher narrativity. Additionally, the R^2 change for each of these features exceeds 0.05, confirming their strong explanatory power for the high non-narrativity in Waley's translation. However, as shown in Table 3, there is no statistically significant difference in the use of PRESP (present participial clauses) between the two translations ($p = 0.734 > 0.05$). Therefore, the primary linguistic features that account for the stronger non-narrativity in Waley's translation are synthetic negation, perfect aspect verbs, third-person pronouns, and public verbs. Synthetic negation (e.g., neither, no, nor) enhances textual clarity by emphasizing specific characteristics, reducing ambiguity, and ensuring a more precise transmission of the author's intent. Consequently, as its frequency increases, narrativity becomes more pronounced. Perfect aspect verbs indicate completed past actions, reinforcing temporal sequencing. Third-person pronouns refer to animate entities, typically humans, distinguishing referents from the speaker and addressee. Public verbs (e.g., admit, assert, declare, hint, report, say) signal indirect or reported speech (Biber 1988: 109). Table 3 further reveals that Waley's translation employs synthetic negation and

public verbs less frequently while using perfect aspect verbs and third-person pronouns more extensively, ultimately resulting in a lower degree of narrativity.

The five linguistic features that most significantly contribute to the higher narrativity in Pound's translation are SYNE, PRESP, PUBV, PEAS, and TPP3, collectively accounting for 99.5% of the variance in this dimension ($R^2 = 0.995$). The standardized regression equation is: Dimension 2 (Pound's translation) = -0.444 + 0.991SYNE + 0.999PRESP + 1.026PUBV + 1.120PEAS + 1.005TPP3. Among these features, the R^2 change for synthetic negation (SYNE) and present participial clauses (PRESP) exceeds 0.05, indicating their stronger explanatory power for the heightened narrativity in Pound's translation compared to public verbs, perfect aspect verbs, and third-person pronouns. However, as shown in Table 5.3, there is no statistically significant difference in the use of present participial clauses (PRESP) between Pound's and Waley's translations ($p = 0.734 > 0.05$). Therefore, synthetic negation emerges as the most influential factor in explaining Pound's higher narrativity. Further analysis in Table 3 confirms that Pound's translation employs synthetic negation significantly more frequently than Waley's, reinforcing its stronger narrative quality.

Example 2

ST: 旱既大甚，则不可沮。赫赫炎炎，云我无所。大命近止，靡瞻靡顾。群公先正，则不我助。

WT: The drought is long and deep, It cannot be curtailed. It is parching, a burning heat; We have **no** place to escape, The hand of fate closes near, There is none to look to, none to care. The fonner ministers and their lords, Even they do not give us aid.

PT: The great drought! None can withstay it, It is impetuous fire against which I have **no** recourse. The great destiny draws to an end, we find **neither** awe **nor** shelter, **nor** do the pastoral lords of aforetime bring us their aid,

It is an example demonstrating that Pound's translation employs significantly more synthetic negation than Waley's, illustrating a fundamental stylistic divergence between the two translators. The original poem describes King Xuan of the Zhou dynasty praying for rain.

Waley's translation remains faithful to the original while incorporating a strong philosophical dimension. For instance, in his rendering, the line "It is parching, a burning heat; We have no place to escape" not only conveys a literal depiction of drought but also evokes a broader existential struggle. The imagery of "parching" and "burning heat" extends beyond physical suffering to suggest spiritual desolation, resonating with the notion of a disrupted cosmic order. This dual-layered interpretation highlights Waley's ability to embed philosophical depth within a faithful representation of the source material.

In contrast, Pound's translation amplifies emotive intensity and dramatic expression through the use of synthetic negation. For example, the sentence "we find neither awe nor shelter, nor do the pastoral lords of aforetime bring us their aid" employs multiple instances of negation to reinforce a profound sense of alienation, emphasizing an individual's isolation from both divine and earthly sources of support. This stylistic choice heightens the translation's dramatic tone, immersing the reader in

an existential crisis. While this approach enhances the text's emotional impact and broadens its appeal to modern audiences, it also risks diverging from the original poem's communal and cosmological themes. The Zhou dynasty's prayer for rain, which reflects collective suffering and a plea for cosmic harmony, is transformed in Pound's version into a deeply individualistic lament—an interpretation shaped more by his own literary sensibilities than by the ethos of the source text.

4.2.3 Contrast of On-Line Informational Elaboration

Dimension 6 differentiates translations based on their degree of on-line information elaboration, where higher values indicate a more informational nature, often produced under time constraints, similar to speeches. This dimension initially comprises nine linguistic features: THAC, DEMO, TOBJ, THVC, STPR, EX, DEMP, WHOBJ, and PHC. However, several features are excluded due to their distribution across multiple dimensions or low statistical significance:

- EX and DEMP exhibit loads below 0.35 across all seven dimensions.
- STPR has a higher loading on dimension 1.
- WHOBJ and PHC load more heavily on dimension 3.
- The mean Z-scores of THAC and TOBJ fall below 1.

As a result, only two linguistic features—DEMO and THVC—are retained for the calculation of dimension 6.

Stepwise regression analysis was conducted in SPSS on the linguistic feature DEMO for both Waley's and Pound's translations in dimension 6, as presented in Tables 8 and 9.

Table 8 Parameters of the Stepwise Regression Models for the Top Variable Predicting the Scores of Dimension 6 of Waley's and Pound's Translations

Model	R	R Square	Adjusted R Square	Std. Error of the Estimate
Waley's Translation	0.872	0.760	0.760	2.20906
Pound's Translation	0.792	0.628	0.626	1.67689

Table 9 Stepwise Regression Coefficients for Dimension 6 of Waley's and Pound's Translations

Model		Unstandardized Coefficients		Standardized Coefficients	t	p
		B	Standard Error	Beta		
Waley's Translation	(Constant)	-0.627	0.128		-4.888	0.000
	DEMO	1.024	0.033	0.872	31.008	0.000
Pound's Translation	(Constant)	-0.699	0.105		-6.658	0.000
	DEMO	1.033	0.046	0.792	22.594	0.000

The regression results indicate that the most significant linguistic feature explaining the scores on dimension 6 for both Waley's and Pound's translations is the use of demonstrative pronouns (DEMO). Demonstrative pronouns account for 76.0% of the score in Waley's translation (R^2 = 0.760) and 62.8% in Pound's translation (R^2 = 0.628). The standardized regression equations are as follows: Dimension 6 (Waley's

translation) = -0.627 + 1.024DEMO; Dimension 6 (Pound's translation) = -0.699 + 1.003DEMO. The positive regression coefficients suggest that a higher frequency of demonstrative pronouns correlates with a more informational text. As cohesion in unplanned informative discourse largely depends on demonstrative pronouns (Biber 1988: 114), their increased use enhances textual cohesion. Cross-referencing with Table 3, it is evident that Waley's translation employs significantly more demonstrative pronouns than Pound's, which explains why Waley's translation exhibits stronger on-line information elaboration.

Example 3

ST: 有狐绥绥，在彼淇梁。心之忧矣，之子无裳。有狐绥绥，在彼淇厉。心之忧矣，之子无带。有狐绥绥，在彼淇侧。心之忧矣，之子无服。（国风·魏风·有狐）

WT: There is a fox dragging along By that dam on the Ch'i. Oh, my heart is sad; **That** man of mine has no robe. There is a fox dragging along By **that** ford on the Ch'i. Oh, my heart is sad; **That** man of mine has no belt. There is a fox dragging along By **that** side of the Ch'i. Oh, my heart is sad; **That** man of mine has no coat.

PT: K'i dam, prowls fox, a heart 's to hurt and someone's there has got no skirt. By the K'i's deep on the prowl; got no belt on, bless my soul. Tangle-fox by K'i bank tall: who says: got no clothes at all?

This example illustrates that Waley's translation employs demonstrative pronouns more frequently than Pound's. The original poem, written from a woman's perspective, laments her husband's destitution and lack of clothing as he wanders (Cheng and Jiang, 2017: 145).

Waley's translation prominently features the demonstrative pronoun "that", using it in two distinct contexts: to refer to objects and to denote locations. First, "that" is employed to specify "之子" in the original poem, as seen in Waley's translation of "之子" as "That man of mine." This usage makes the reference more explicit, clarifying relationships between characters, reducing the reader's cognitive load, and enhancing the translation's readability. Second, "that" is used to indicate specific locations, such as "that dam," "that ford," and "that side," corresponding to "淇梁", "淇厉", and "淇侧" in the original text. This strategy effectively localizes the poem's geographic setting, allowing readers to visualize the scenes more intuitively and strengthening the poem's spatial coherence.

By contrast, Pound's translation prioritizes vivid visual imagery, focusing on both the portrayal of the fox and the depiction of the environment. First, Pound conveys the fox's mysterious and agile nature through dynamic verbs like "prowl" and "tangle", reinforcing a sense of movement and enigma. This enhances the poetic atmosphere, making the scene more immersive. Second, Pound enriches the poem's imagery by vividly depicting the landscape. Phrases such as "By K'i's deep" and "by K'i bank tall" evoke the depth of the Qi River and the steepness of its banks, amplifying the poem's visual impact. The interplay between the fox's elusive movements and the detailed environmental descriptions creates a heightened sense of mystery and vitality. Additionally, Pound incorporates colloquial expressions like "bless my soul," which injects a conversational tone, further enlivening the translation.

5. Disscusion on the Causes of Register Differences

To further analyze the register differences between Waley's and Pound's translations, this study explores the ideological, poetic, and patronage factors influencing their composition, framed within the context of manipulation theory.

5.1 Contrast of Ideology

Arthur Waley and Ezra Pound, two influential figures in literary translation, approached their translation of *Shijing* with contrasting ideologies, shaping their methodologies and the registers of their respective works.

Initially, Waley undervalued *Shijing*, claiming that out of the three hundred and five poems, only about thirty are likely to engage the modern reader. He believed the majority of the poems focused on war or love and that their beauty could not be replicated in English (Waley 1997: 26). In his preface to *The Temple and Other Poems*, Waley argued that Chinese folk-songs were originally collected for political purposes and misinterpreted by Confucian scholars as ethical documents, with love songs often being seen as allegories of the ruler-minister relationship (Waley 1923: 10). Waley's political views aligned with those of the Bloomsbury Group, and his anti-imperialist stance became more pronounced after World War I, particularly in advocating for the return of looted Chinese artworks to China (Chen 2010: 75). His encounter with the work of scholars like Hu Shi and Gu Jiegang, who applied Western methods to study Chinese history, further influenced his views, leading him to see *Shijing* as no longer sacred (Xiong 2007: 118). However, after reading Marcel Granet's anthropological analysis of *Shijing*, Waley redefined it as a reflection of primitive Chinese social life, translating the title as *Book of Songs* rather than *Book of Poetry*. Waley's ideological shift impacted the register of his translation, focusing on the social and cultural contexts behind the poems. His use of analytical negation, first-person pronouns, and modal verbs indicating possibility enhanced interactivity, allowing readers to engage emotionally with the text. Additionally, the use of demonstrative pronouns helped structure the poems, facilitating a clearer understanding of their content.

In contrast, Pound viewed *Shijing* primarily through its moral and educational function, perceiving Confucianism as a remedy for the moral decay of Western civilization. For him, translating *Shijing* was not just an academic exercise but a political act aimed at using Confucian teachings to restore social order and moral responsibility in the West (Zhou and Zhou 2023: 2-4). Pound's translation, titled *The Confucian Odes: The Classic Anthology Defined by Confucius*, reflects his deep respect for Confucian thought and its potential to address the moral crises of the West. His interpretation of the odes, especially those praising rulers, aligned with his ideological commitment to totalitarianism. In particular, King Wen is presented as a symbol of centralized power, with phrases like "King Wen's law is our right" reinforcing his belief in authoritarian rule (Li 2009: 91). Unlike Waley, who emphasized the cultural and social dimensions of *Shijing*, Pound's translation was politically charged, aiming to use Confucianism to restore moral order in Western society. Thus, the ideological commitments of both translators shaped not only the

content but also the registers and impact of their translations. Pound's ideological stance deeply influenced the registers of his translation, which is marked by strong informativeness and narrativity. His conviction in Confucianism's transformative power led him to emphasize clarity and precision in conveying key concepts. This is evident in his frequent use of nouns and prepositional phrases, which deliver substantive meaning while avoiding unnecessary embellishment. Furthermore, Pound's use of synthetic negations and public verbs enhances the narrative quality of his translation, constructing a cohesive and compelling narrative. This narrative structure underscores the significance of Confucian principles, presenting them as a coherent ideological framework to address the moral challenges facing Western society.

5.2 Contrast of the Poetics

Arthur Waley and Ezra Pound embody two distinct translation philosophies in their English renderings of classical Chinese poetry, each reflecting their unique poetics.

Waley, influenced by the modernist poetry movement, departed from the Victorian tradition and rejected the use of English poetic metrics for translating classical Chinese poetry, instead advocating for free verse. In his translation of *Shijing*, Waley's poetics are primarily reflected in his commitment to fidelity to the original text. His attention to detail and his dedication to capturing the nuances of classical Chinese poetry are evident throughout his work. Notably, Waley's translations stand out for their non-Orientalist approach, distinguishing him from many Orientalist scholars influenced by Eurocentric views, which often led to the beautification or distortion of Eastern texts (Jiang 2008: 15). Waley, by contrast, maintained a respectful and egalitarian attitude toward "other" cultures, aiming for "literal translation, not free translation" (Waley 1919: 33). Xu Yuanchong (1991: 35) categorized early translators of Chinese poetry into three groups: the literalists (e.g., Waley), paraphrasts (e.g., Herbert Allen Gile), and imitators (e.g., Ezra Pound). Waley's poetics significantly shape the register of his translation. For instance, in his rendering of the poem *采薇*, he translates the line "靡室靡家，玁狁之故．不遑启居，玁狁之故" (No house, no home, nomadic tribes' reason. Not at ease, settle, nomadic tribes' reason) as: "We have no house, no home Because of the Hsien-yun. We cannot rest or bide Because of the Hsien-yun." Waley directly translates "玁狁" (a northern ethnic group in ancient China and an enemy of the Zhou dynasty) to "Hsien-yun," and adds the subject "we" to emphasize the agentive case, creating a straightforward English sentence structure of subject + predicate + object. This clarity and simplicity have contributed to the enduring popularity of Waley's translations, which are widely appreciated by English readers and have become canonical in the field (Liang 2015: 91-92).

Ezra Pound's poetics, defined by energy, emotion, and precision, offer a distinct approach to translation. For Pound, energy in poetry captures the power of tradition, race consciousness, and cultural association. In *How to Read*, Pound categorizes poetic energy into three forms: Melopoeia (sound and rhythm), Phanopoeia (imagery),

and Logopoeia (intellectual atmosphere). Pound's use of Logopoeia revitalizes *Shijing* by emphasizing the nuanced interplay between words, transcending their direct meanings. In his translation of 采薇, for instance, he translates "采薇采薇，薇亦作止" as "Pick a fern, pick a fern, ferns are high," creating a dynamic flow that mirrors the original's energy. This contrasts with Waley's more literal translation, which adds narrative elements. Emotion is central to Pound's poetics, guiding both form and content (Yuan 2012: 26-27). He argues that emotions should shape word choice, infusing poetry with authenticity. In his 采薇 translation, the phrase "Home', I'll say: home, the year's gone by" invites readers to feel the poet's emotions, blending homesickness with the passage of time. Pound's personal emotional connection deepens the poem's emotional resonance, especially during his internment. In addition, Pound also emphasizes precision, using words that directly convey meaning while eliminating excess (Yuan 2012: 28). His translation of *采薇* is more concise than Waley's, by omitting the subject and simplifying syntax, focusing on the action itself, thus creating a direct and impactful translation. His deliberate omission of lines like "忧心烈烈..." enhances the poem's rhythm and thematic clarity, highlighting precision as key to emotional depth.

References

Biber, D. 1988. *Variation across Speech and Writing* [M]. Cambridge: Cambridge University Press.

Chen, Hui. 2010. A Study on Arthur Waley's Translation [D]. Ph.D. Dissertation. Hunan Normal University.

Chen, Meishan. 2021. Is courtroom discourse an "oral" or "literate" register? the importance of sub-register [J]. *Discourse Studies* 23(3): 249-273.

Cheng, Junying & Jianyuan Jiang. 2017. *Commentary on Book of Songs* [M]. Beijing: Zhonghua Book Company.

Clarke, I. 2022 A multi-dimensional analysis of English tweets [J]. *Language and Literature* 31(2): 124-149.

Garrote, 2021. Macroestructura de correos electrónicos de petición en el contexto académico. análisis contrastivo español-italiano [J]. *Onomazein* (52): 127-142.

Halliday, M. 1988. On the language of physical science [A]. In M. Ghadessy (ed.). *Register of Written English: Situational Factors and Linguistic Features* [C]. London: Pinter. 162-178.

Hu, Chunyu & Jinlin Tan. 2020. A multi-dimensional analysis of register features of letters to shareholders by Chinese and American enterprises [J]. *Foreign Languages and Their Teaching* (6): 66-75.

Jack, G. 2023. Register variation explains stylometric authorship analysis [J]. *Corpus Linguistics and Linguistic Theory* 19(1): 47-77.

Jiang, Jinlin & Jiajin Xu. 2015. A corpus-based multi-dimensional analysis of business English register [J]. *Foreign Language Teaching and Research* 47(2): 225-236.

Jiang, Xiaohua. 2008. Orientalism and translation: a re-consideration [J]. *Chinese Translators Journal* (5): 11-18.

Li, Guangwei. 2021. On the English Translation and Dissemination of *Shijing* or *The Book of Poetry* from the Perspective of Hermeneutics [D]. Ph.D. Dissertation. Fujian Normal University.

Li, Yuliang. 2009. The "present" principle and imagism in Pound's translation of *Shijing* [J]. *Foreign Language Education* (3): 90-94.

Liang, Zhenhui. 2015. Arthur Waley's humanism underlying his translation of Chinese poetry [J]. *Foreign Language Learning Theory and Practice* (1): 89-93.

Ma, Zuyi & Rongzhen Ren. 2003. *History of World's Translation of Chinese Wrings* [M]. Wuhan: Hubei Education Press.

Nini, A. 2019. The Multi-Dimensional Analysis Tagger [A]. In T. Sardinha & M. Pinto (eds). *Multi-Dimensional Analysis: Research Methods and Current Issues* [C]. London & New York: Bloomsbury Academic. 67-94.

Poole, R. 2021. A corpus-aided study of stance adverbs in judicial opinions and the implications for English for legal purposes instruction [J]. *English for Specific Purposes* 62(2): 117-127.

Ryoo, M. & D. Cao. 2021. A multi-dimensional analysis of maritime English registers [J]. *English Language Teaching* 33(1): 1-25.

Waley, A. 1919. *A Hundred and Seventy Poems* [M]. New York: Alfred A. Knopf, Inc.

Waley, A. 1923. *The Temple and Other Poems* [M]. London: George Allen & Unwin Ltd.

Waley, A. 1997. *A Hundred and Seventy Chinese Poems* [M]. Sandwich: Chapman Billies.

Wang, Hong, Jie Shen, Cui Wang & Xingfeng Liu. 2021. *A Study on the New Development of English Translation of Chinese Classics* [M]. Beijing: Tsinghua University Press.

Wang, Wei. 2021. A corpus-based multidimensional analysis of the register features of English TED talks [J]. *Foreign Language Education* 42(2): 23-28.

Wang, Zuoliang & Wangdao Ding. 1987. *Introduction to English Stylistics* [M]. Beijing: Foreign Language Teaching and Research Press.

Xiong, Wenhua. 2007. *History of Sinology in Britain* [M]. Beijing: Academy Press.

Xu, Yuanchong. 1991. Development of verse translation [J]. *Journal of Foreign Languages* (1): 35-45.

Yang, Linlin & Lingli Zhong. 2018. A corpus-based multidimensional analysis of business hearing registers: a case study of Facebook's joint hearing [J]. *Foreign Languages and Translation* 25(3): 52-58.

Yuan, Jing. 2012. A Study on Ezra Pound's Translation of *Shi Jing* [D]. Ph.D. Dissertation. Zhejiang University.

Zhao, Chaoyong. 2020a. Genre variations in *Jin Ping Mei*'s two full English translation: a corpus-based multidimensional analysis [J]. *Foreign Language Teaching and Research* 52(2): 283-295.

Zhao, Chaoyong. 2020b. Using corpus-based multidimensional analysis for the comparative description of translator's style [J]. *Foreign Language Learning Theory and Practice* (3): 67-73.

Zhou, Ruowen & Debo Zhou. 2023. Reasons for the transformation of Pound's

translation of *Shijing* [J]. *Journal of University of Shanghai for Science and Technology*.
