# Supplementary material for: Multidimensional analysis of register variation in English translations of Shijing
Source: PLoS One. 2025 Sep 18;20(9):e0332503. doi: 10.1371/journal.pone.0332503 (PMC12445467; doi:10.1371/journal.pone.0332503)
Supplement: Attachment — Submitted filename: Response to Reviewers.docx [file pone.0332503.s003.docx]

**A rebuttal letter**

Dear Editor and Reviewers,

We sincerely thank you and the reviewers for your valuable feedback on our manuscript. We have carefully considered all the comments and revised the manuscript accordingly. Below, we outline how we have responded to each of the required and recommended changes.

**Required Changes for Acceptance:**

1. **Terminology Alignment with MDA Framework: The manuscript must revise its use of the term "register" to align with Douglas Biber's definition in the context of Multi-Dimensional Analysis (MDA). Reviewer 1 rightly highlights conceptual inconsistencies that undermine the study's methodological foundation. The term must be applied accurately, and the authors should avoid conflating MDA with cluster analysis or stylistic variation outside the MDA framework.**

Response: We sincerely thank the reviewer for this valuable comment regarding our use of the term register and our description of MDA. We have carefully revised the manuscript to ensure that the terminology aligns with Biber's framework.

In the revised Introduction, we now define register following Biber & Conrad (2009) as "a variety associated with a particular situation of use (including particular communicative purposes)." We explicitly explain that, within the MDA framework, register is operationalized as a functional language variation characterized by co-occurring linguistic features that reflect communicative goals and situational contexts. We also emphasize that register variation is not limited to vocabulary but involves multiple interacting linguistic features.

Revised passage (Introduction):

"The concept of register is central to this study. It refers to 'a variety associated with a particular situation of use (including particular communicative purposes)' (Biber & Conrad, 2009, p. 6). Within the framework of Multi-Dimensional Analysis (MDA), a register is operationalized as a functional language variation characterized by co-occurring linguistic features that reflect specific communicative goals and situational contexts. … By applying MDA to the translations of *Shijing*, we can examine how features such as modality, interactivity, and information structure vary across the two translators' works."

We corrected the inaccurate statement that previously conflated MDA with cluster analysis. The text originally read:

"MDA, developed by Douglas Biber, uses factor analysis and cluster analysis to identify and calculate the frequency and standardized frequency per thousand words of sixty-seven linguistic features."

This has been revised to:

"MDA, developed by Douglas Biber, applies factor analysis to identify sixty-seven linguistic features. It also calculates their frequency and standardized frequency per thousand words. "

This correction reflects the fact that cluster analysis is not part of MDA as defined by Biber. MDA uses factor analysis (not cluster analysis) to identify dimensions of co-occurring linguistic features. Cluster analysis is a distinct statistical technique often applied in text typology or classification, but it is not included in the MDA method.

The rest of the paper has been adjusted accordingly.

We believe these revisions resolve the conceptual inconsistencies noted by the reviewer and now present a clear and accurate application of Biber's MDA framework.

2. **Clarify Theoretical Justification and Method: The manuscript must explicitly clarify why MDA was selected as the method, and how it has been correctly applied to poetry translation. The theoretical basis (Biber's framework) must be better explained and directly linked to the research questions and linguistic features analyzed.**

Response: We appreciate the reviewer's suggestion to clarify the theoretical justification and methodological application of MDA in the context of poetry translation. We have revised the Introduction and Section 3.3 (Tools and Methods) to make these connections explicit.

In the Introduction, we added a passage that explains why MDA is particularly suitable for this study. MDA systematically identifies patterns of co-occurring linguistic features, making it a rigorous framework for uncovering subtle functional variations in texts. Since poetry translation involves a complex interplay of cultural, stylistic, and ideological factors, MDA provides a principled way to quantify how linguistic choices contribute to broader communicative goals.

Revised passage (Introduction):

"The suitability of MDA for this study stems from its ability to systematically identify and quantify the subtle differences in how translators' linguistic choices contribute to the broader communicative goals of the text. As poetry translation involves complex decisions that balance cultural, stylistic, and ideological considerations, MDA offers a rigorous framework to uncover these nuances."

In Section 3.3, we now explicitly link the use of MDA to our research questions. We highlight how the sixty-seven linguistic features analyzed through MDA provide insights into dimensions. These are directly relevant to examining how Waley's and Pound's translations of *Shijing* reflect different thematic orientations and communicative purposes.

Revised passage (Section 3.3):

"This combined approach is particularly well-suited to studying translation register, as it goes beyond impressionistic or qualitative judgments. By relying on statistically derived dimensions and rigorous testing, it enables systematic comparison of linguistic features across translations and reveals register-level patterns, such as differences in modality, interactivity, and information structure. They are directly relevant to our research questions, since these patterns illuminate how Waley's and Pound's translations of *Shijing* reflect distinct thematic orientations and communicative purposes."

We believe these revisions strengthen the theoretical foundation of the study and clarify the methodological rationale for using MDA in the analysis of poetry translation.

3. **Data Availability Compliance: Although a GitHub link is provided, the authors must ensure that all linguistic data supporting their statistical claims are fully accessible and described in the Data Availability Statement, in line with PLOS ONE's policy.**

Response: We thank the reviewer for pointing out the importance of ensuring compliance with PLOS ONE's data availability policy. We have revised the Data Availability Statement to explicitly confirm that all linguistic data supporting the statistical analyses are fully accessible.

Specifically, we updated the Figshare repository to include:

English translations of *Shijing* by Arthur Waley and Ezra Pound, and the analysis results of the two translations by MAT 1.3.3, including 67 linguistic feature counts and dimension scores for six dimensions.

The data underlying the results presented in the study are available from https://doi.org/10.6084/m9.figshare.29890838.v1

The dataset description now reads:

"This item includes three compressed packages. Two of the packages, named 'text files,' contain the English translations of *Shijing* by Arthur Waley and Ezra Pound, respectively. Each package contains 305 text files. Since the original *Shijing* consists of 305 chapters, the translations have been organized accordingly. Researchers can unzip these packages and import the entire folder into Multidimensional Analysis Tagger 1.3.3 (or a newer version) for analysis. The package, named 'linguistic feature counts and dimension scores,' contains the analysis results of the two translations by MAT 1.3.3, including 67 linguistic feature counts and dimension scores for six dimensions. Researchers can import these results into SPSS for further analysis, thereby replicating the research findings."

We believe this revision ensures full compliance with PLOS ONE's data sharing requirements and enhances the transparency and reproducibility of the study.

4. **Language and APA Style Compliance: Substantial editorial revisions are required to improve clarity and consistency. This includes correcting grammatical issues, simplifying complex sentence structures, and ensuring APA-style in-text citations and references (e.g., changing "Wang et al., 2021: 67" to "Wang et al., 2021, p. 67").**

Response: We thank the reviewer for this important reminder regarding clarity and style compliance. We have carefully revised the manuscript to address these issues.

We conducted a thorough editorial review of the manuscript to correct grammatical errors, simplify overly complex sentence structures, and improve overall readability. Where appropriate, we divided long sentences into shorter units to ensure clarity.

We systematically reviewed all in-text citations and references to ensure full adherence to the APA 7th edition guidelines. Similarly, the reference list was carefully checked for consistency in capitalization, italics, and punctuation.

We believe these editorial and formatting revisions have substantially improved the clarity, consistency, and professional presentation of the manuscript.

**Recommended Changes (not required for acceptance but strongly advised):**

1. **Expand Literature Review with Greater Thematic Coherence: The current literature review reads as a list. Reorganize thematically (e.g., by domain or method) and integrate more directly with the research objective, particularly in the underrepresented area of poetry translation using MDA.**

Response: We thank the reviewer for highlighting the need for greater thematic coherence in the literature review. In response, we have substantially revised Section 2 as follows:

The literature review has been reorganized into two thematic strands: (a) research on register variation in technical and professional translation, and (b) research on register variation in literary translation, with particular attention to underrepresented work on using MDA for poetic texts.

Each strand now explicitly connects prior work to the research questions of the present study. For example, we highlight how previous MDA studies identify specific linguistic features relevant to register analysis, and we note the gap in applying MDA to poetry translation, which directly motivates our investigation of Waley's and Pound's translations of *Shijing*.

We have smoothed transitions between studies to avoid a "list-like" presentation, providing critical commentary on how each study informs the present work, rather than simply summarizing prior literature.

These revisions strengthen the conceptual foundation of our study and clarify how our research contributes to the underexplored area of poetry translation using MDA.

2. **Strengthen Integration of Textual Examples: While statistical results are sound, the manuscript would benefit from more illustrative examples that make the differences between Waley and Pound's styles tangible. Several reviewers recommend including quotations that exemplify the highlighted features.**

Response: We thank the reviewers for the suggestion to include more illustrative examples. In response, we have added two additional examples (Example 2 and Example 3) to more clearly demonstrate the stylistic contrasts between Waley and Pound.

For Example 1, in the previous version we focused on noun usage; however, the differences between the two translations were not sufficiently marked. We revised this example to highlight first-person pronouns, which show a more pronounced divergence in how Waley and Pound handle narrative perspective and emotional engagement. Waley's frequent use of "I" and "we" foregrounds personal experience and reader interactivity, while Pound's translation minimizes first-person reference, emphasizing concise, informative narration.

Examples 2 and 3 further illustrate specific linguistic contrasts: Example 2 highlights Waley's reliance on possibility modals versus Pound's emphatic negation, and Example 3 shows Waley's consistent use of analytic negation compared with Pound's more descriptive, narrative strategy. Together, these examples make the differences in register between the two translators more tangible and complement the statistical analyses presented in the manuscript.

3. **Discussion Section Improvements: Reviewers suggest summarizing the ideological and poetic differences at the end of each subsection (5.1, 5.2) to clearly link them back to the observed register distinctions. Consider adding a brief reference to postcolonial or Orientalist frameworks to enrich the ideological discussion, particularly regarding Pound's interpretation.**

Response: We are grateful for this constructive suggestion. In the revised Discussion (Section 5), we have substantially strengthened the connection between ideological/poetic orientations and the register profiles of Waley's and Pound's translations. Specifically, we now conclude each subsection (5.1 and 5.2) with a summary that explicitly links ideological and poetic differences to the identified register distinctions, ensuring thematic coherence and analytical clarity.

Moreover, we have incorporated a reference to Orientalist frameworks (Said, 2003) to contextualize Pound's interpretation. This addition highlights how his moralizing and politically instrumentalized stance risks reproducing an Orientalist dynamic, contrasting with Waley's anthropological and anti-imperialist orientation.

In addition, we have introduced a new subsection (5.3) on patronage. Although patronage was found to exert limited influence on register formation compared with ideology and poetics, this section further situates the translations within their socio-historical publishing contexts. By adding 5.3, we present a more comprehensive account of the constraints outlined in manipulation theory, thereby enriching the overall explanatory framework of the Discussion.

These revisions both broaden and deepen the section, ensuring that the register analysis is firmly situated within linguistic, cultural, and socio-historical perspectives.

In addition, other suggestions raised by the reviewers and editor have also been addressed and marked in the main text.

We appreciate the constructive feedback and have made extensive revisions to the manuscript based on the reviewers' and editor's suggestions. We believe the revisions significantly improve the clarity, coherence, and methodological rigor of the study, addressing all of the concerns raised. We look forward to your feedback on the revised manuscript and hope that it will now meet PLOS ONE's publication criteria.

Thank you for your consideration.

Sincerely,

Li Baohu, Li Guangwei
